# Evaluation of the discrepancy between clinical diagnostic hypotheses and anatomopathological diagnoses resulting from autopsies

**DOI:** 10.6061/clinics/2019/e1197

**Published:** 2019-09-10

**Authors:** Talita Zerbini, Julio M Singer, Vilma Leyton

**Affiliations:** I Departamento de Medicina Legal, Etica Medica e Medicina Social e do Trabalho, Faculdade de Medicina FMUSP, Universidade de Sao Paulo, Sao Paulo, SP, BR; II Instituto de Matematica e Estatistica, Universidade de Sao Paulo, Sao Paulo, SP, BR

**Keywords:** Autopsy, Medical Eerrors, Cause of Death, Diagnosis

## Abstract

**OBJECTIVES:**

An objective of clinical autopsies is to determine the final cause of death and the pathological changes that may have triggered it. Despite advances in Medicine, the level of discrepancy between clinical and autopsy diagnoses remains significant. The aim of this study was to compare the data obtained from autopsies carried out at the São Bernardo do Campo/SP Death Verification Section with clinical diagnostic hypotheses proposed during medical care.

**METHOD:**

This was a retrospective study involving the comparison of necroscopic reports issued by the São Bernardo do Campo/São Paulo Death Verification Section in 2014 and 2015 and the Cadaver Referral Guides completed by attending physicians prior to the necroscopic examination.

**RESULTS:**

A total of 465 cases were analyzed. In general, discrepancies between the clinical diagnostic hypothesis and the autopsy diagnosis occurred in 28% of the cases. A logistic regression model, with diagnostic discrepancy as a response variable and sex, age, duration of care, type of institution providing medical care and organ system as explanatory variables, was fit to the data; the results indicated that all explanatory variables with the exception of organ system are not significant (*p*>0.132).

**CONCLUSIONS:**

Discrepancies between clinical diagnostic hypotheses and autopsy diagnoses continue to occur, despite new developments in complementary examinations and therapies. The odds of a discrepancy when patients present with diseases of the cardiac system are greater than those when there are problems in the vascular, endocrine and neurological systems.

## INTRODUCTION

Autopsies are traditionally useful for improving the quality of health care, as the conclusions obtained in the associated exams provide complementary information on the diseases, thus allowing improvement in the quality of the therapy that can be offered, in the quality control of the provided care, and in the access to technological innovations in laboratory studies 1,2. Originally, autopsies were scientific examinations of corpses in which the whole body and all organs were exposed and examined to determine the cause of death and the related circumstances 3. In Brazil, these tests can be performed in cases of violent, suspicious or natural death. Violent death is the result of an external and harmful action, regardless of whether it is immediate or delayed; a suspicious death is one that presents the possibility of being caused by a violent action, usually occurring suddenly and without evident cause. Natural deaths are due to morbid processes that are not related to exogenous factors 4. According to Ordinance No. 116 published by the Brazilian Ministry of Health on February 11, 2009, the bodies of people who died due to natural causes without medical assistance or with a poor diagnosis of the *causa mortis* should be referred to the Death Verification Section for clinical autopsy. According to Mateos et al. 5, an objective of clinical autopsies is to determine the final cause of death and the pathological changes that may have triggered it.

Errors in medical diagnosis are erroneously treated as impossible within the health system 6, because of technological developments and the high expectations among the population regarding the accuracy of medical work due to extensive exposure in the media 7. Professionals in this field may be subject to administrative, civil and even criminal charges when he or she cannot establish a precise medical diagnosis. However, the variables that underlie medical diagnoses are numerous and difficult to characterize, rendering the decision susceptible to error. Thus, the first step in reducing diagnostic errors is to encourage awareness of professionals and of the population about the real possibility of their occurrence 8. Despite advances in Medicine, the level of discrepancy between clinical and autopsy diagnoses is estimated to be 10% to 20% of the cases 9. Therefore, it is critical that autopsies continue to be performed in order to detect possible failures in diagnostic processes and to seek tools to minimize them.

In view of the relevance of the topic and the importance of the comparison between the clinical diagnostic hypotheses and anatomopathological diagnoses obtained by autopsy, we aimed to compare data obtained in autopsies performed at the São Bernardo do Campo/São Paulo Death Verification Section with diagnostic hypotheses proposed during medical care. It is important to note that the city has only one specialized referral center for autopsies in cases of natural death; therefore, the analysis of the institution's data reliably reflects the general panorama of the pathology service available in the city.

According to the revised scientific literature and the authors' knowledge, there are only few published Brazilian studies related to possible diagnostic discrepancies, none of which examined cases in São Bernardo do Campo. We provided an expanded view of autopsied cases in the city with a record of previous care, with the objective of providing the health department with knowledge that may be employed to improve health care quality.

## MATERIALS AND METHODS

This was a retrospective study aimed at comparing diagnoses obtained in two different situations: medical care and autopsy. A total of 465 necroscopic reports issued by the São Bernardo do Campo/São Paulo Death Verification Section between 2014 and 2015 were analyzed along with the Cadaver Referral Guides completed by the attending physicians prior to the necroscopic examination.

There were no exclusion criteria due to anthropometric characteristics of the cadavers, such as age, sex, weight and height. Cases that had incomplete documentation, such as the absence or incomplete Cadaver Referral Guides, were excluded. In addition, cases in which death occurred without physician care immediately prior to death (such as deaths in residences or public places) were also excluded from the study since this would preclude a comparative analysis of health care and pathological data.

The type of institution was categorized according to the city's organizational system, since the health department classifies all health care institutes according to infrastructure, taking into account the availability of specialized staff and technological resources. The groups were categorized to avoid analysis bias due to structural differences among the institutions.

The time that each patient took to reach the health care institute was not analyzed because of its subjective nature (patients that did not arrive in the health care unit in an ambulance, for instance). In addition, the time of care variable refers only to the last hospitalization prior to death.

We considered all the clinical hypotheses that have been registered by the attending physicians. The diseases were categorized according to the respective organ system and there were no cases with more than one organ system hypothesis.

The autopsy diagnosis refers to the immediate cause of death, since the clinical hypothesis specified by the attending physician in the Cadaver Referral Guides is also related to the immediate cause of death. All autopsies were performed by medical pathologists with at least 10 years of experience and all the cases were revised by at least two pathologists to avoid any mistakes in the determination of the cause of the death.

According to the Goldman criteria, the discrepancies can be classified into four different categories, namely, class I (missed major diagnosis with a potential adverse impact on survival and that would have changed management) and class II (missed major diagnosis with no potential impact on survival and that would have not changed therapy), which are considered major discrepancies, and class III (missed minor diagnosis related to terminal disease but not related to the cause of death) and class IV (other missed minor diagnosis), which are minor discrepancies 10. We considered only major discrepancies, given that minor discrepancies are not of interest to the public health department.

Data were stored in a spreadsheet with information on sex, age (years), time (h) between the beginning of care at the clinic and death, type of health facility (Emergency Care Units [ECUs] or hospital), clinical diagnosis (and organ system) obtained by the physician responsible for care and the corresponding diagnosis obtained from the pathologist responsible for the autopsy.

To analyze the data, we used a logistic regression model, with diagnostic discrepancy as the response variable and sex, age, duration of care, type of institution and organ system diagnosed by the attending physician as explanatory variables.

### Ethics

The research was approved by the research ethics committee (report number 1,954,123), and the authors were exempt from obtaining Free and Informed Consent.

## RESULTS

A descriptive analysis of the data indicated a slight male predominance in the sample (53%). In addition, 59% of the bodies were referred from second and third level health unit services (hospitals) and 41% were referred from first level health unit services (basic units). Age was grouped according to published articles with similar themes (Table 1). The same type of grouping was considered for the duration of care (Table 2).

The most frequent diagnosis assigned by the attending physician was acute myocardial infarction (AMI), followed by sepsis (inflammatory reaction secondary to the presence of an infectious focus). The frequency of diagnoses is shown in Table 3.

The clinical diagnoses were grouped into organ systems (e.g., cardiac, digestive, respiratory). The frequency of the organ systems, according to the assisting physician's diagnosis, is shown in Table 4.

The most frequent diagnosis suggested by the pathologist was AMI, followed by pulmonary thromboembolism (PTE) and bronchopneumonia (Table 5).

The diagnoses were grouped according to the same organ systems used in the analysis of the attending physicians' diagnoses. The frequencies of diagnoses according to organ system based on the pathologist's diagnosis resulting from the autopsy are indicated in Table 6.

The joint distribution of frequencies of the clinical diagnoses and autopsy diagnoses is provided in Table 7.

The cardiac system presented the highest diagnostic agreement among all the organ systems. In general, a discrepancy between the clinical diagnosis and the autopsy diagnosis occurred in 28% of the cases (Table 8).

A logistic regression model 11 with diagnostic discrepancy as a response variable and sex, age, duration of care, type of institution and organ system as explanatory variables was fit to the data and indicated that no explanatory variables, except organ system, were significant (*p*>0.132). One case involving the lymphatic system (where there was agreement) and two cases involving the urinary system (where there was disagreement) were eliminated from the analysis to improve the fit of a model in which only the diagnosis-associated system was considered as the explanatory variable. The model can be represented as:

log(Odds of discrepancy) = A + B(i) x Diagnostic system(i)

where A corresponds to the odds of diagnostic discrepancy for the cardiac system and B(i) is the odds ratio between the diagnostic discrepancy for the system i and the cardiac system (i=1: infectious, i=2: respiratory, i=3: digestive, i=4: neurological, i=5: endocrine and i=6: vascular).

According to this model, the odds of discrepancy and confidence intervals with a confidence coefficient of approximately 95% are shown in Table 9.

## DISCUSSION

The general discrepancy rate between the clinical diagnoses and the autopsy diagnoses was 28%, similar to those published in Spain (25.6%) 12 and England (28%) 13. Studies in other localities revealed higher rates, such as in the United States (44%) 14, or lower rates, such as in the Netherlands (18.1%) 15 or Switzerland (7%) 16.

Our results indicate that only the diagnosis-related system is associated with the discrepancy between the clinical diagnosis and the autopsy diagnosis. This is in agreement with results published by Fares et al. 17, Aalten et al. 18 and Kotovicz et al. 19. However, some previously published articles stated that the discrepancy is related to the shorter duration of care 15,20,21 and sex and age differences 14,15.

Descriptively, the cardiac system presented the highest diagnostic agreement, with low odds of diagnostic discrepancy (0.172), followed by the infectious system (0.443) and respiratory system (0.463); this outcome is in agreement with previously published studies, and according to Kotovicz et al. 19, AMI, PTE and pneumonia diagnoses rarely present diagnostic discrepancy. In light of this result, it is possible to conclude that health institutions are prepared to perform cardiac diagnoses. However, the odds of discrepancy for the vascular (2.333), endocrine (2.000) and neurological (1.500) systems were extremely high, which is also in agreement with previous studies indicating that the vascular system presents the greatest odds of discrepancy 22. Thus, it is essential that attending physicians broaden the range of diagnostic possibilities at the time of care, remembering the possibility of aneurysm dissection, ruptured aneurysms and strokes, which were associated with greater probabilities of discrepancy.

It is important to emphasize that the complexity of health care institutions were not associated with diagnostic discrepancy rates, as the values corresponding to first level (basic units) and second and third levels (hospitals) were similar. According to Espinosa-Brito et al. 23 and Kuijpers et al. 15, the use of complementary exams or new technologies has not been able to reduce diagnostic discrepancy rates, clearly demonstrating that the physician's most powerful diagnostic tool is his or hers semiology. One of the pillars of medicine is the semiological examination, which may make the request for complementary exams unnecessary in some situations. For example, a well-performed anamnesis provides correct clinical diagnoses in approximately 60% of the cases; when combined with the physical examination, the accuracy increases to nearly 80% 24.

Our study has several limitations, including the retrospective study design that relies on documentation that was completed by different physicians (attending physicians and pathologists). In addition, given that an autopsy is usually requested when the cause of death remains uncertain, major discrepancies are more likely to be identified, possibly leading to a higher incidence of discrepancy levels. The fact that there were only 2 endocrine and only 10 vascular cases as opposed to 208 cardiac cases is another drawback but this is reflected in the size of the corresponding confidence intervals, which are much wider for the former. Finally, since the study was limited to cases from only one city, it is difficult to determine how generalizable the results are.

## CONCLUSIONS

Discrepancies between clinical diagnoses and autopsy diagnoses continue to occur, despite the progress of complementary examinations and therapies. In this study, discrepancy occurred in 28% of the analyzed cases, with lower odds of discrepancy in patients with diseases of the cardiac system and greater odds of discrepancy in patients with problems of the vascular, endocrine and neurological systems. Thus, it is essential that the attending physician perform a thorough semiotechnical examination during care so that he or she can consider the range of diagnostic possibilities.

## AUTHOR CONTRIBUTIONS

Zerbini T was responsible for the study conception and design, data collection and manuscript drafting. Singer JM was responsible for the data analysis. Leyton V was responsible for the manuscript drafting.

## Figures and Tables

**Table 1:** Frequencies by age.

Age	Frequency	Relative frequency (%)
0-14	17	4
15-24	6	1
25-34	14	3
35-44	31	6
45-54	59	13
55-64	93	20
65-74	101	22
75+	144	31
Total	465	100

**Table 2:** Frequencies by duration of care.

Duration of care (h)	Frequency	Relative frequency (%)
0-1.0	130	28
1.1-5.0	106	23
5.1-36.0	113	24
36.1+	116	25
Total	465	100

**Table 3:** Frequencies of clinical diagnoses hypotheses.

Clinical diagnoses hypothesis	Frequency	Relative frequency (%)
Acute abdomen	6	1.3
Metabolic acidosis	3	0.6
Ruptured aortic aneurysm	2	0.4
Cardiac arrhythmia	24	5.2
Hemorrhagic stroke	13	2.8
Ischemic stroke	8	1.7
Bronchoaspiration	15	3.2
Bronchopneumonia	14	3.0
Bronchiolitis	1	0.2
Pancreatic carcinoma	1	0.2
Carcinomatosis	1	0.2
Dilated cardiomyopathy	2	0.4
Hypertensive cardiomyopathy	1	0.2
Ischemic heart disease	2	0.4
Diabetic ketoacidosis	3	0.6
Cardiogenic shock	23	4.9
Hypovolemic shock	6	1.3
Mixed shock	2	0.4
Neurogenic shock	2	0.4
Refractory shock	2	0.4
Aorta dissection	1	0.2
Chronic obstructive pulmonary disease	1	0.2
Acute pulmonary edema	16	3.4
Hepatic encephalopathy	1	0.2
Hypoxic encephalopathy	1	0.2
Epilepsy	3	0.6
Rocky Mountain spotted fever	1	0.2
Alveolar hemorrhage	1	0.2
Upper GI bleeding	13	2.8
Incisional bleeding	1	0.2
Hepatitis	1	0.2
Intracranial hypertension	1	0.2
Pulmonary hypoplasia	1	0.2
Hypoxia	1	0.2
Subarachnoid hemorrhage	2	0.4
Acute myocardial infarction	115	24.7
Jaundice	1	0.2
Surgical site infection	1	0.2
Liver failure	7	1.5
Kidney failure	2	0.4
Respiratory failure	25	5.3
Mesenteric ischemia	3	0.6
Leptospirosis	2	0.4
Lymphoma	1	0.2
Abdominal mass	1	0.2
Meningitis	5	1.1
Meningococcemia	1	0.2
Meningoencephalitis	1	0.2
Metastasis	1	0.2
Pulmonary metastasis	1	0.2
Biliary neoplasia	1	0.2
Esophageal neoplasia	1	0.2
Pneumonia	4	0.9
Sepsis	77	16.6
Neonatal sepsis	2	0.4
Consumptive syndrome	1	0.2
Cardiac tamponade	1	0.2
Traumatic brain injury	2	0.4
Pulmonary thromboembolism	30	6.5
Coronary thrombosis	1	0.2
Pulmonary thrombosis	1	0.2
Tuberculosis	1	0.2
Total	465	100

**Table 4:** Frequencies of diagnoses per organ system.

Organ system	Frequency	Relative frequency (%)
Cardiac	184	39.6
Digestive	36	7.7
Endocrine	6	1.3
Infectious	88	18.9
Lymphatic	1	0.2
Neurological	40	8.6
Respiratory	98	21.1
Urinary	2	0.4
Vascular	10	2.2
Total	465	100

**Table 5:** Autopsy diagnoses frequencies.

Diagnoses	Frequency	Relative frequency (%)
Acute abdomen	4	0.9
Brain abscess	1	0.2
Metabolic acidosis	1	0.2
Anencephaly	1	0.2
Aortic dissection	7	1.5
Ruptured aortic aneurysm	7	1.5
Pulmonary atelectasis	1	0.2
Hemorrhagic stroke	12	2.6
Bronchoaspiration	3	0.6
Bronchodysplasia	1	0.2
Bronchopneumonia	45	9.7
Infected bronchiectasis	1	0.2
Bronchiolitis	1	0.2
Carcinomatosis	1	0.2
Dilated cardiomyopathy	6	1.3
Hypertrophic cardiomyopathy	1	0.2
Ischemic heart disease	12	2.6
Biliary cirrhosis	1	0.2
Hepatic cirrhosis	5	1.1
Diffuse alveolar damage	4	0.9
Hyaline membrane disease	2	0.4
Chronic obstructive pulmonary disease	4	0.9
Acute pulmonary edema	44	9.5
Brain edema	7	1.5
Tuberculoid encephalitis	1	0.2
H1N1 infection	1	0.2
Hemoperitoneum	1	0.2
Upper GI bleeding	11	2.4
Hydrocephalus	1	0.2
Intracranial hypertension	2	0.4
Pulmonary hypoplasia	1	0.2
Acute myocardial infarction	150	32.3
Pulmonary infarction	1	0.2
Influenza A	1	0.2
Heart failure	3	0.6
Liver failure	3	0.6
Respiratory failure	2	0.4
Mesenteric ischemia	5	1.1
Leptospirosis	1	0.2
Lymphoma	1	0.2
Meningitis	1	0.2
Hepatic necrosis	3	0.6
Pulmonary malignant neoplasm	1	0.2
Hemorrhagic pancreatitis	1	0.2
Necrotizing papillitis	1	0.2
Pericarditis	1	0.2
Acute peritonitis	3	0.6
Pneumonia	9	1.9
Sepsis	37	8.0
Cardiac tamponade	4	0.9
Pulmonary thromboembolism	47	10.1
Total	465	100

**Table 6:** Frequencies of autopsy diagnoses per organ system.

Organ system	Frequency	Relative frequency (%)
Cardiac	208	44.7
Digestive	37	8.0
Endocrine	2	0.4
Infectious	64	13.8
Lymphatic	1	0.2
Neurological	25	5.4
Respiratory	113	24.3
Vascular	15	3.2
Total	465	100

**Table 7:** Organ system diagnoses distribution.

Clinical diagnoses	Diagnosis on autopsy								
	Cardiac	Digestive	Endocrine	Infectious	Lymphatic	Neurological	Respiratory	Vascular	Total
Cardiac	157	3	0	0	0	2	14	8	184
Digestive	5	25	0	0	0	0	6	0	36
Endocrine	1	1	2	0	0	0	2	0	6
Infectious	10	3	0	63	0	0	12	0	88
Lymphatic	0	0	0	0	1	0	0	0	1
Neurological	12	0	0	0	0	17	8	3	40
Respiratory	18	4	0	1	0	5	68	2	98
Urinary	1	1	0	0	0	0	0	0	2
Vascular	4	0	0	0	0	1	3	2	10
Total	208	37	2	64	1	25	113	15	465

**Table 8:** Discrepancy frequencies between the clinical and autopsy diagnoses.

Discrepancy	Frequency observed	Relative frequency (%)
No	334	72
Yes	131	28
Total	465	100

**Table 9:** Discrepancy odds and 95% confidence intervals.

Diagnosis-related system	Odds of discrepancy	Confidence interval (95%)	
		Lower limit	Upper limit
Cardiac	0.172	0.114	0.259
Infectious	0.443	0.281	0.696
Respiratory	0.463	0.289	0.708
Digestive	0.500	0.250	1.000
Neurological	1.500	0.797	2.824
Endocrine	2.000	0.366	10.920
Vascular	2.333	0.114	9.025

## References

[1] Ladich E, Burke A, Virmani R (2006). Should the autopsy be allowed to become obsolete. Nat Clin Pract Cardiovasc Med.

[2] Lundström C, Persson A, Ross S, Ljung P, Lindholm S, Gyllensvärd F (2012). State-of-the-art of visualization in post-mortem imaging. APMIS.

[3] Patowary AJ (2012). Virtopsy: the non traumatic autopsy. NE Quest.

[4] França GV (2004). Medicina legal.

[5] Mateos FPA, Fernández FÁ, Fernández MM, Román JG, Val Bernal JF (2009). La autopsia cl&iacute;nica. Revista Electrónica de Autopsia.

[6] Grade MH, Zucoloto S, Kajiwara JK, Fernandes MT, Couto LG, Garcia SB (2004). Trends of accuracy of clinical diagnoses of the basic cause of death in a university hospital. J Clin Pathol.

[7] Sanders R (2003). Medical technology: a critical perspective. The Internet Journal of Medical Technology.

[8] Lester H, Tritter JQ (2001). Medical error: a discussion of the medical construction of error and suggestions for reforms of medical education to decrease error. Med Educ.

[9] Rodrigues FR, Lopes VG, Lopez CL, Soares Filho PJ, Silva RC, Silva LE (2011). O decr&eacute;scimo vertiginoso das aut&oacute;psias em um hospital universit&aacute;rio do Brasil nos &uacute;ltimos 20 anos. J Bras Patol Med Lab.

[10] Pastores SM, Dulu A, Voigt L, Raoof N, Alicea M, Halpern NA (2007). Premortem clinical diagnoses and postmortem autopsy findings: discrepancies in critically ill cancer patients. Crit Care.

[11] Hosmer DW, Lemeshow S, Sturdivant RX (2013). Applied logistic regression.

[12] Val-Bernal JF (2015). [The current role of autopsy in current clinical practice]. Med Clin.

[13] Mosquera DA, Goldman MD (1993). Surgical audit without autopsy: tales of the unexpected. Ann R Coll Surg Engl.

[14] Gibson TN, Shirley SE, Escoffery CT, Reid M (2004). Discrepancies between clinical and postmortem diagnoses in Jamaica: a study from the University Hospital of the West Indies. J Clin Pathol.

[15] Kuijpers CC, Fronczek J, van de Goot FR, Niessen HW, van Diest PJ, Jiwa M (2014). The value of autopsies in the era of high-tech medicine: discrepant findings persist. J Clin Pathol.

[16] Schwanda-Burger S, Moch H, Muntwyler J, Salomon F (2012). Diagnostic errors in the new millennium: a follow-up autopsy study. Mod Pathol.

[17] Fares AF, Fares J, Fares GF, Cordeiro JA, Nakazone MA, Cury PM (2011). [Clinical and pathological discrepancies and cardiovascular findings in 409 consecutive autopsies]. Arq Bras Cardiol.

[18] Aalten CM, Samson MM, Jansen PA (2006). Diagnostic errors; the need to have autopsies. Neth J Med.

[19] Kotovicz F, Mauad T, Saldiva PH (2008). Clinico-pathological discrepancies in a general university hospital in S&atilde;o Paulo, Brazil. Clinics.

[20] Zhu K, Feng H, Xu Y, Mao Z, Zhang W, Chen J (2014). An analysis of 60 years of autopsy data from Zhejiang university in Hangzhou, China. PLoS One.

[21] Tavora F, Crowder CD, Sun CC, Burke AP (2008). Discrepancies between clinical and autopsy diagnoses: a comparison of university, community, and private autopsy practices. Am J Clin Pathol.

[22] Winters B, Custer J, Galvagno SM, Colantuoni E, Kapoor SG, Lee H (2012). Diagnostic errors in the intensive care unit: a systematic review of autopsy studies. BMJ Qual Saf.

[23] Espinosa-Brito AD, de Mendoza-Amat JH (2017). In Defense of Clinical Autopsy and Its Practice in Cuba. MEDICC Rev.

[24] Rodrigues AN, Cunha CS, Cunha CS, Neto JO, Souza MT (2011). A semiologia m&eacute;dica no s&eacute;culo XXI. Cadernos UniFoa.

